# The impact of structured counselling on patient knowledge at a private TB program in Karachi

**DOI:** 10.12669/pjms.36.ICON-Suppl.1713

**Published:** 2020-01

**Authors:** Sheikh Sulaiman Sajjad, Nabila Sajid, Asad Fatimi, Nawal Maqbool, Naila Baig-Ansari, Farhana Amanullah

**Affiliations:** 1 Sheikh Sulaiman Sajjad, A Levels Student, Karachi Grammar School, Karachi, Pakistan; 2 Nabila Sajid, Psychosocial Counsellor, The Indus Hospital TB Program, The Indus Hospital, Korangi Crossing, Karachi, Pakistan; 3 Asad Fatimi, A Levels Student, Karachi Grammar School, Karachi, Pakistan; 4 Nawal Maqbool, A Levels Student, Karachi Grammar School, Karachi, Pakistan; 5 Naila Baig-Ansari, PhD, Chair Indus Hospital Research Center, The Indus Hospital, Korangi Crossing, Karachi, Pakistan; 6 Farhana Amanullah, MBBS, DABP, FAAP. Senior Consultant, Pediatrics, The Indus Hospital, Korangi Crossing, Karachi, Pakistan

**Keywords:** Treatment adherence, TB counselling, TB prevention

## Abstract

**Objective:**

To assess the impact of structured counselling on the knowledge of patients and families attending the Tuberculosis (TB) clinic at the Indus Hospital, Karachi.

**Methods:**

This was a case control study conducted from 17th December 2018 to 28th December 2018 at The Indus Hospital, Karachi. We evaluated the baseline knowledge regarding TB in 60 patients and families, 30 of whom had undergone at least one counselling session at the TB clinic. We then compared the scores achieved by each group in three main categories of tuberculosis: disease, treatment and prevention.

**Results:**

The average scores in all three categories of TB knowledge were higher in counselled participants compared to non-counselled participants.

**Conclusion:**

We found that structured counselling resulted in improved patient knowledge and clarified common misconceptions about TB which has been shown to result in improved patient outcomes. Effective counselling is an easy to implement strategy in a low resource setting. A trained psychosocial counsellor is essential for every TB program in Pakistan.

## INTRODUCTION

TB is the ninth leading cause of death worldwide and the leading cause from a single infectious agent, ranking above HIV/AIDS. In 2017, 10 million people were estimated to have developed TB disease, of which 5.8 million were men, 3.2 million women and 1 million children.^1^ TB claims nearly 4500 lives a day. It impacts not only health and well-being, but also society and economy due to loss in work productivity, absenteeism, ongoing transmission and premature death.^1^-^3^ In Pakistan, TB claims an estimated 54 thousand people lives and there are over 500000 new cases annually, representing a third of the TB burden.^4^ The Pakistan National TB program provides vertical TB service outlets but these are poorly integrated with primary healthcare and are not patient centric. They offer no structured counselling or social support to TB affected families suffering income losses and transport costs. Meanwhile, in the private sector there is no standardised TB treatment or counselling at all.^5^

Effective treatment of Tuberculosis requires multiple medications daily for months to years, depending on the level of drug resistance. Failure to complete prescribed therapy can lead to poorer outcomes such as treatment failure, disease relapse, further disease transmission, drug resistance and death.^6^ Poor treatment adherence remains a major obstacle to eradication of TB in high burden countries.^7^,^8^ Directly observed therapy (DOT) by community health workers, although associated with better adherence outcomes in one meta-analysis.^9^ had limited benefit when studied in Pakistan.^10^ A study in Sialkot in 1999 reported improved outcomes with intensive counselling in TB patients.^11^ There are no recent studies from Pakistan or other high TB burden countries assessing the individual role of counselling and patient education, a cost effective strategy compared to DOT, in improving awareness and attitude towards disease.

The TB program at The Indus Hospital (TIH) offers focused disease-based counselling and mental health assessments to TB patients and affected families. We conducted this study to assess changes in understanding and perspectives of patients and families through structured counselling. This may lead to better treatment outcomes, patient empowerment and reduction of stigma. This study is unique as it systematically assesses knowledge improvement to highlight the importance of structured counselling, a relatively low cost intervention in a low resource setting where other strategies such as DOT, financial social support, video assisted technologies etc. are difficult to implement.

## METHODS

This was a case control study involving patients and their families who attended the outpatient TB clinic of TIH, a free-of-charge tertiary facility located in a low-income industrial area of Karachi. The catchment population exceeds 2.5 million people and 350+ patients are seen in the TB clinic daily.

The Indus Hospital TB Program, established in 2008, is located in a purpose built airborne infection control setting. Services include a full time psychologist and psychosocial counsellor. Each patient diagnosed with TB receives counselling on disease and treatment as well as adherence, infection control measures and contact screening conducted through structured verbal counselling as well as wall charts and brochures. The psychosocial component includes discussing and dispelling myths and misconceptions regarding TB and any patient specific psycho/behavioural counselling. A patient mental health assessment is performed (using a validated screening tool for Pakistani population AKU-ADS).^12^ Monthly visits with the psychologist, ensuring social support, adherence and management of side-effects are conducted in conjunction with the clinical visits.

The study was conducted from 17th December 2018 to 28th December 2018. The sample size was calculated based on earlier pilot surveys which identified participants with knowledge and understanding about TB (81.9 vs 43.4), its treatment duration (100 vs 52.1), TB in children (100 vs 63.6), treatment plan (100 vs 43.4), preventive measures against TB (72.8 vs 47.8) and importance of measures against TB (36.4 vs 17.4) with and without counselling respectively. Sample size was set at 60 using WHO sample size determination software with 95% CI, 6.5% level of significance, 80% power and a one-sided test type. The participants were the families attending the TB clinic, out of which 30 had been previously counselled, by a doctor, a counsellor (or both), and the remaining 30 were attending the TB clinic but had not received any structured counselling yet.

Inclusion criteria for this study was patients newly diagnosed with any form of TB and registered for treatment at the Indus Hospital TB Program. Those transferred in from other facilities, still undergoing workup and/or not yet registered in the program were excluded.

### Ethical considerations

Prior institutional IRB approval from IRD-IRB of TIH was obtained (IRD_IRB_2018_08_008). No patient identifiers were collected and a phone number of the study principal investigator was provided if the patient/family had any additional questions or concerns regarding the study.

The data collection tool used was a questionnaire with 23 questions. The first question determined whether they had or had not received clinic counselling. Other demographics collected included respondents age and if they were the patient or if they were the guardian or family member of the patient. The knowledge assessment questions were designed in a true-false format to gauge participant’s knowledge of three main aspects: TB disease, TB treatment and TB prevention. Each section had five questions and a correct answer equalled to a score of one. The questionnaire also collected feedback about patient satisfaction with clinic facilities.

### Statistical analysis

Data were entered and analysed using SPSS version 24.0. Mean ± SD or Median (IQR) was computed as appropriate for knowledge scores. Independent sample T-test/Mann-Whitney U test was applied as appropriate to assess difference in knowledge scores between to counselled and non-counselled participants. ANOVA/Kruskal-Wallis test was applied as appropriate to assess difference in knowledge scores among type of counsellors. Chi-square test was applied to assess association between knowledge questions and counselled and non-counselled participants.

## RESULTS

A total of 60 participants were enrolled in this study of which 30 had received counselling and 30 were non-counselled, 37 (45%) were males and the median age was 29.5 years. In the counselled group, 13 (43%) participants had been counselled by a counsellor, 8 (27%) by a doctor and 9 (30%) participants by both. Among the participants, 30 (50%) had TB themselves, 18 (30%) were relatives of TB patients and 12 (20%) were the parents of children with TB.

Almost all participants knew that TB is curable. The study showed that counselled participants had higher knowledge scores of TB disease, treatment and prevention than the non-counselled participants (Table-I).

**Table I:** Difference in knowledge scores according to counselling status.

Knowledge scores	Counselling status						P-value

	Counselled n=30			Non Counselled n=30			

	Mean± SD	Min-Max	Median (IQR)	Mean ± SD	Min-Max	Median (IQR)	
TB disease	4.13±0.8	2-5	4(4-5)	3.0±1.0	1-5	3(2-4)	0.000**
TB Treatment	4.7±0.64	2-5	5(5-5)	3.8±1.2	1-5	4(3-4)	0.000**
TB prevention	4.3±0.64	3-5	4(4-5)	3.8±0.71	2-5	34-5)	0.000**

** p-value<0.0001, Mann-Whitney U test.

We found that knowledge scores were slightly better if counselling was done by a doctor and highest if counselled by both counsellor and doctor, however there was no significant difference in knowledge about TB treatment and prevention based on the type of counsellor (Table-II). No statistically significant difference was found between the knowledge scores of patients themselves, relatives of TB patients and parents of a child with TB (Table-II).

**Table II:** Difference in knowledge scores among types of counsellors and participants.

	TB disease	TB Treatment	TB prevention
*Types of counsellors*			
*Doctor*			
Mean ± SD	4.3± 0.5	4.7± 0.46	4.0± 0.53
Min-max	4-5	4-5	3-5
Median (IQR)	4(4-5)	5(4-5)	4(4-4)
Counsellor			
Mean ± SD	3.8± 0.98	4.6± 0.86	4.2± 0.68
Min-max	2-5	2-5	3-5
Median (IQR)	4(3-4)	5(4-5)	4(4-5)
Both			
Mean ± SD	4.3± 0.7	4.8± 0.33	4.6± 0.50
Min-max	3-5	4-5	4-5
Median (IQR)	4(4-5)	5(5-5)	5(4-5)
*Total*			
Mean ± SD	4.13±0.82	4.73±0.64	4.27±0.64
Min-max	2-5	2-5	3-5
Median (IQR)	4(4-5)	5(5-5)	4(4-5)
*P- value*	0.250^ɫ^	0.718^†^	0.062^†^
*Types of participant*			
Self			
Mean ± SD	3.6±1.0	3.9±1.14	3.87±0.78
Min - Max	2-5	1-5	2-5
Median (IQR)	4(3-4)	4(3-5)	4(3-4)
Child			
Mean ± SD	3.42±1.2	4.4±1.2	3.9±0.93
Min - Max	2-5	1-5	2-5
Median (IQR)	3.5(2-4.7)	5(4.2-5)	4(3-4.7)
Relative			
Mean ± SD	3.7±1.02	4.2±1.0	3.7±0.83
Min - Max	1-5	2-5	2-5
Median (IQR)	4(3-4)	4(3-5)	4.5(3.8-4)
*Total*			
Mean ± SD	3.60±1.0	4.1±1.13	3.8±0.8
Min - Max	1-5	1-5	2-5
Median (IQR)	4(3-4)	3.3(4.5-5)	4(3-4)
*P- value*	0.806^ɫ^	0.221^†^	0.839^ɫ^

ɫ one-way ANOVA.

† Kruskal Wallis test.

Further analysis of the detailed knowledge differences between the counselled and non-counselled cohort showed that although the baseline knowledge of the non-counselled participants was good, some misconceptions about mode of transmission TB and types of TB (Table-III) were found in this group that were not present in the counselled cohort. Structured counselling led to recipients more likely to know that TB was caused by a germ/bacteria compared to non-counselled (p=0.001), can involve organs other than lungs (p=0.02) and had better knowledge about signs and symptoms suggestive of TB (p=0.001). The counselled cohort knew that medicines had to be taken on an empty stomach (p<0.001) and that no dietary restrictions were needed during treatment (p<0.001). Regarding transmission and prevention, the counselled cohort was more likely to know that spread did not occur by sharing utensils (p=0.002) and that open windows and good ventilation prevents TB transmission (p= 0.002) (Table-III).

**Table III:** Differences in detailed knowledge between the counselled and non-counselled participants.

	Counselled			P-value

	Yes	No	Total
*Disease Knowledge*				
*Caused by bacteria*				
Right	19 (63.3)	6b (20)	25 (41.7)	0.001*
Wrong	11 (36.7)	24b (80)	35 (58.3)
Curable				
Right	30 (100)	29 (96.7)	59 (98.3)	1.000
Wrong	30 (100)	29 (96.7)	59 (98.3)
*Can involve organs other than lungs*				
Right	25 (83.3)	17 (56.7)	42 (70)	0.024*
Wrong	5 (16.7)	13 (43.3)	18 (30)
*High Risk groups are vulnerable*				
Right	21 (70)	20 (66.7)	41 (68.3)	0.781
Wrong	9 (30)	10 (33.3)	19 (31.7)
*Knowledge about signs and symptoms of disease*				
Right	29 (96.7)	19 (63.3)	48 (80)	0.001*
Wrong	1 (3.3)	11 (36.7)	12 (20)
*TB treatment Knowledge*				
*Treatment Duration*				
Right	29 (96.7)	23 (76.7)	52 (86.7)	0.052
Wrong	1 (3.3)	7 (23.3)	8 (13.3)
*Use program meds only*				
Right	29 (96.7)	27 (90)	56 (93.3)	0.612
Wrong	1 (3.3)	3 (10)	4 (6.7)
*Treatment Adherence*				
Right	29 (96.7)	24 (80)	53 (88.3)	0.103
Wrong	1 (3.3)	6 (20)	7 (11.7)
*Medicine to be taken on empty stomach*				
Right	30 (100)	19 (63.3)	49 (81.7)	0.000**
Wrong	0 (0)	11 (36.7)	11 (18.3)
*Special Diet Not Essential*				
Right	25 (83.3)	10 (33.3)	35 (58.3)	0.000**
Wrong	5 (16.7)	20 (66.7)	25 (41.7)
TB Prevention Knowledge				
Airborn				
Right	24 (80)	19 (63.3)	43 (71.7)	0.152
Wrong	6 (20)	11 (36.7)	17 (28.3)
*Cough Hygiene*				
Right	29 (96.7)	30 (100)	59 (98.3)	1.000
Wrong	1 (3.3)	0 (0)	1 (1.7)
*Not Spread by Utensils*				
Right	18 (60)	6 (20)	24 (40)	0.002*
Wrong	12 (40)	24 (80)	36 (60)
*Windows Open to reduce spread*				
Right	28 (93.3)	18 (60)	46 (76.7)	0.002*
Wrong	2 (6.7)	12 (40)	14 (23.3)
*Small children & prevention*				
Right	30 (100)	30 (100)	60 (100)	
Wrong	0(0)	0(0)	0(0)	

* P value<0.05,

** P-value<0.0001, Chi-Square Test.

The TB program facilities feedback indicated 83% participants were satisfied with the services. Of the 10 who were not completely satisfied most cited busy clinics and wait times as the main problem.

## DISCUSSION

TB remains a global problem and is the leading cause of death from a single infectious pathogen, despite the existence of effective medicines for over half a century. TB is known to be an extremely contagious disease and prompt diagnosis and treatment are required to stop transmission in the community. Low-income families are disproportionately affected often due to densely populated living conditions with poor access to quality services. Poor compliance and loss to follow-up add to the medical and social cost of TB and produce drug resistance and ongoing disease transmission.

Structured counselling and social support are important components of quality TB services. A study from Peshawar showed good treatment outcomes in pulmonary TB patients who received structured counselling.^13^ In India, higher mean post-test knowledge scores and improved overall outcomes among TB patients who received intensive health education was seen.^14^ A recent meta-analysis of observational studies concluded that TB treatment outcomes are improved with the use of adherence interventions such as patient education and counselling, incentives and enablers, as well as reminders.^9^

We found that most families attending the Indus Hospital (TIH) TB clinic had some knowledge about TB at baseline. This is a reflection of the TB program’s community based awareness raising work since inception in 2008. A multifaceted TB case detection strategy was implemented by the Indus Hospital TB program in the surrounding population in 2011.^15^ This included an intense one-year long TB awareness raising campaign and general practitioner (GP) engagement that resulted in a significant increase in TB detection and had far reaching community wide epidemiological impact.^16^ Over 50 GPs had been engaged in TB diagnosis and management. The Zero TB initiative in Karachi began TB detection efforts in 2016, further strengthening awareness in the Korangi/Landhi area served by TIH.^17^ These public awareness messages may be the reason we found that almost everyone interviewed was aware that TB is curable; and only two participants admitted to facing any kind of stigma. This contrasts with findings from a study in Faisalabad, Pakistan, where >80% patients with TB had depression and 25% reported stigma as the underlying cause.^18^

This study is unique as it used a comprehensive tool in a true/false format that evaluated important TB concepts in detail. Our results show that counselling is significantly beneficial in improving patient knowledge and behaviour in all three aspects: disease, treatment and prevention. We found that baseline knowledge was highest about disease treatment which likely reflects the impact of earlier community and GP engagement efforts and the endemic nature of TB in the communities around TIH.

We found that when doctors counselled families, they had better knowledge about disease. This could be because clinicians focus more on disease course and prognosis and were able to impart this knowledge more efficiently. This by no means undermines the effectiveness of the counsellor in enhancing patient’s overall knowledge and providing structured support. It also further points towards the need to disseminate more specific information during counselling sessions, building upon basic understanding that tuberculosis patients may already have. We found maximal improvement in knowledge of disease following counselling (score increased from 3 to 4), but despite this the level of knowledge was still the lowest of the three categories. Although there was improvement in knowledge in all aspects reviewed in depth, we did not find a statistically significant change in knowledge regarding curability of TB, high risk groups, treatment duration, importance of using program medications and adherence, cough hygiene and preventive therapy following counselling. This is likely because of high pre-existing awareness acquired from prior TB communications campaigns, community awareness sessions and even discussions in the waiting area with other patients.

We found that structured counselling specifically helped enhance participant knowledge about signs and symptoms of TB and other organ involvement and clarified concepts about disease transmission. TB patients and their families are known to be important agents of change in the community, so it is imperative that they do so armed with accurate knowledge to help eliminate common TB myths and misconceptions that can delay health seeking and promote stigmatization.^19^ TB affected families help raise community awareness and enable others to reach early diagnosis, treatment and prevention.

## CONCLUSION

Structured counselling improved knowledge about all important detailed aspects of tuberculosis over and above pre-existing knowledge of TB and its curability amongst participants attending the Indus TB clinic. The incorporation of trained counsellors giving disease-specific information to families affected by TB in all TB programs in Pakistan is urgently needed. This will enable TB affected families to act as agents of change, remove stigma, common misconceptions and promote treatment adherence, completion and disease prevention through an effective low cost strategy.

### Author`s contributions

**SSJ:** Contributed to conception, design and implementation of study as well as data collection, and manuscript writing.

**NS:** Contributed to counselling of families and implementation of questionnaire.

**AF:** Contributed to consent form design and translation.

**NM:** Contributed to design and data collection of pilot study.

**NBA:** Contributed to questionnaire development, critical input in intellectual content and data analysis.

**FA:** Contributed to study design, data analysis and manuscript writing.
